# Sand fly population dynamics and cutaneous leishmaniasis among soldiers in an Atlantic forest remnant in northeastern Brazil

**DOI:** 10.1371/journal.pntd.0005406

**Published:** 2017-02-27

**Authors:** Filipe Dantas-Torres, Kamila Gaudêncio da Silva Sales, Débora Elienai de Oliveira Miranda, Fernando José da Silva, Luciana Aguiar Figueredo, Fábio Lopes de Melo, Maria Edileuza Felinto de Brito, Maria Sandra Andrade, Sinval Pinto Brandão-Filho

**Affiliations:** 1 Department of Immunology, Aggeu Magalhães Institute, Oswaldo Cruz Foundation (Fiocruz), Recife, Pernambuco, Brazil; 2 Department of Parasitology, Aggeu Magalhães Research Center, Oswaldo Cruz Foundation (Fiocruz), Recife, Pernambuco, Brazil; 3 Department of Nursing, University of Pernambuco, Recife, Pernambuco, Brazil; Institut de recherche pour le developpement, FRANCE

## Abstract

Outbreaks of cutaneous leishmaniasis are relatively common among soldiers involved in nocturnal activities in tropical forests. We investigated the population dynamics of sand flies in a military training camp located in a remnant of Atlantic rainforest in northeastern Brazil, where outbreaks of cutaneous leishmaniasis have sporadically been described. From July 2012 to July 2014, light traps were monthly placed in 10 collection sites, being nine sites located near the forest edge and one near a sheep and goat stable. Light traps operated from 5:00 pm to 6:00 am, during four consecutive nights. *Leishmania* infection in sand flies was assessed using a fast real-time PCR assay. Cases of cutaneous leishmaniasis among soldiers were also investigated. In total, 24,606 sand flies belonging to 25 species were identified. Males (n = 12,683) predominated over females (n = 11,923). Sand flies were present during all months, being more numerous in March (n = 1,691) and April 2013 (n = 3,324). *Lutzomyia choti* (72.9%) was the most abundant species, followed by *Lutzomyia longispina* (13.8%), *Lutzomyia complexa* (5.3%), representing together >90% of the sand flies collected. Forty cases of cutaneous leishmaniasis were recorded among soldiers from January 2012 to December 2014. *Leishmania* isolates were obtained from eight patients and were all characterized as *Leishmania braziliensis*. Soldiers and anyone overnighting in Atlantic rainforest remnants should adopt preventative measures such as the use of repellents on bare skin or clothes and insecticide-treated tents.

## Author summary

Outbreaks of cutaneous leishmaniasis are relatively common among soldiers involved in nocturnal activities in tropical forests. However, there is limited information on the relationship between sand fly population dynamics and cases of cutaneous leishmaniasis in Atlantic forest remnants. In this study, we investigated the population dynamics of sand flies in a military training camp located in a remnant of Atlantic rainforest in northeastern Brazil, where outbreaks of cutaneous leishmaniasis have sporadically been described. In total, 24,606 sand flies belonging to 25 species were identified. Sand flies were present during all months, being more numerous in March and April 2013. *Lutzomyia choti* was the most abundant species and three pools of females belonging to this species were found to be positive for *Leishmania braziliensis* DNA. Our results suggest that the risk of cutaneous leishmaniasis by *Leishmania braziliensis* in Atlantic rainforest remnants is permanent and thus not dictated by sand fly population peaks. People overnighting in Atlantic rainforest remnants should adopt preventative measures such as the use of repellents on bare skin or clothes and insecticide-treated tents.

## Introduction

Cutaneous leishmaniasis is a neglected tropical disease highly prevalent in Central and South America. Indeed, Brazil, Costa Rica and Peru are among the 10 countries accounting for 70–75% of the global estimated cutaneous leishmaniasis incidence [1]. Only in Brazil, 554,475 cases of cutaneous leishmaniasis were notified to public health authorities between 1988 and 2007, with an average incidence of 17.3 cases per 100,000 inhabitants [1]. The disease is widespread in this country occurring mainly in rural areas and forest environments, affecting mainly individuals older than 10 years and males [1, 2].

While widespread in Brazil, cutaneous leishmaniasis is more prevalent in the Amazon forest and Atlantic forest regions [1]. The Amazon forest biome covers 48% of the Brazilian territory (4,245,024 km^2^), but the Atlantic forest biome has been over explored since the arrival of the first Portuguese settlers and in the past centuries its original cover has been reduced to almost nothing. Today, only 2–5% of its original area is considered to be in its original state (http://www.fao.org/3/a-i3825e/i3825e6.pdf). Indeed, the Atlantic forest region presently encompasses an area of 1,129,760 km^2^, extending along the Atlantic coast of Brazil, from Rio Grande do Norte (in the north) to Rio Grande do Sul (in the south). The largest cities (e.g., Rio de Janeiro and São Paulo) and industries in the country are located in the Atlantic forest region, which houses 70% of the Brazilians and accounts for about 80% of its gross domestic product. Nonetheless, the Atlantic forest biome is still home to about 2,000 species of animals and 20,000 species of plants; a biological diversity similar to that found in the Amazon region (http://www.nature.org/ourinitiatives/regions/southamerica/brazil/placesweprotect/atlantic-forest.xml).

Cutaneous leishmaniasis in the Atlantic forest region usually affects rural and forest workers that live near or literally within forest fragments, as well as people developing nocturnal activities in these environments. For instance, several cases of cutaneous leishmaniasis have been reported among soldiers after periods of nocturnal training activities in remnants of Atlantic forest in northeastern Brazil [3–6]. Indeed, the presence of proven and putative sand fly vectors of parasites such as *Leishmania braziliensis*, the most widespread causative agent of cutaneous leishmaniasis in the Americas, is acknowledged in these areas [3, 7–11].

Nonetheless, our knowledge on the ecology of sand flies (Phlebotomine) and its relationship with cutaneous leishmaniasis incidence in the Atlantic forest region is still fragmentary. In this study, we investigated the sand fly fauna in a military training camp located in a remnant of Atlantic rainforest in northeastern Brazil, where outbreaks of cutaneous leishmaniasis have been sporadically described. Our hypothesis was that the temporal dynamics of sand flies was correlated with climate variables and the incidence of cutaneous leishmaniasis in this region.

## Materials and methods

### Ethics statement

This study used secondary data (i.e., anonymized data that has previously been collected in the course of normal care) on human cutaneous leishmaniasis obtained from Reference Service on Leishmaniasis of the Aggeu Magalhães Institute, Oswaldo Cruz Foundation (Fiocruz). No ethical approval was required.

### Study sites

**Fig 1:**
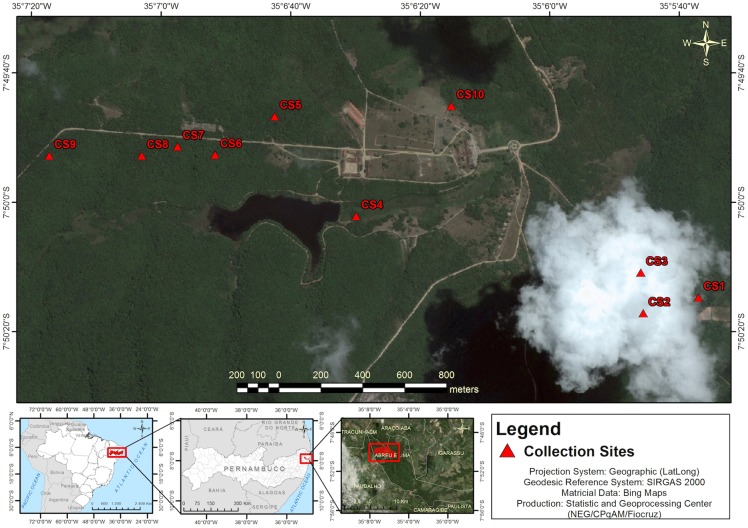
Location of the study area.

This study was carried out in the Campo de Instrução Militar Marechal Newton Cavalcanti (CIMNC), located in the Atlantic forest zone of Pernambuco State. This military training camp comprises an area of 7,324 hectares (Fig 1), distributed in five municipalities: Araçoiaba, Paulista, Igarassu, Paudalho and Tracunhaém. The camp possesses has a central pavilion of command, two villages with 16 adjoining houses, a school, a chapel, 14 houses portholes, eight sheds used for any troops under training and six areas for military training and exercises.

The climate of this region is rainy tropical type with dry summer. The vegetation is represented by the Atlantic rainforest distributed into two main types: open ombrophilous forest and seasonal semidecidual. Outbreaks of cutaneous leishmaniasis are sporadically reported in this region [3–5].

### Sand fly collection and identification

Sand fly collections were carried out monthly, for four consecutive nights, from July 2012 to July 2014 in 10 pre-selected collection sites (CS1-CS10), totaling 96 actual nights and 960 total trap-nights (Table 1). In each site, a CDC-type light trap was placed *ca*. 1.5 m above the ground, operating from 5:00 pm to 6:00 am. Collection sites were typically near animal holes, trunks and roots of large trees, usually in shady and humid environments. Collection sites were also near the places where the soldiers use to camp overnight during training periods. The only exception was CS10, which was a sheep and goat stable, located near the military camp headquarter.

**Table 1:** Brief description of collection sites selected in this study.

Collection site	Geographical coordinates	Altitude	Description
CS1	7° 50.25’ S	133 m	Area near the forest edge, with predominant shrubby vegetation and fruit trees; thin litter layer
	35° 05.62’ W		
CS2	7° 50.29’ S	139 m	Interior of the forest, with dense shrubby-arboreal vegetation; thick litter layer
	35° 05.76’ W		
CS3	7° 50.18’ S	162 m	Interior of the forest, with some clearings; presence of a large tingling, animal burrows and decaying trees
	35° 05.77’ W		
CS4	7° 50.04’ S	116 m	Area near the forest edge, with a dam and water streams; thin litter layer
	35° 06.50’ W		
CS5	7° 49.78’ S	174 m	Forest interior, with shrubby-arboreal vegetation, mainly lianas; area used for military training activities
	35° 06.71’ W		
CS6	7° 49.88’ S	178 m	Forest interior, with shrubby-arboreal vegetation; thick litter layer
	35° 06.86’ W		
CS7	7° 49.86’ S	169 m	Forest interior; with shrubby-arboreal vegetation; thick litter layer
	35° 06.96’ W		
CS8	7° 49.88’ S	151 m	Forest interior; with shrubby-arboreal vegetation; thick litter layer; animal burrows and decaying trees
	35° 07.05’ W		
CS9	7° 49.88’ S	173 m	Forest interior; with shrubby-arboreal vegetation; thick litter layer
	35° 07.29’ W		
CS10	7° 49.75’ S	125 m	Sheep and goat stable, located in an open area near the military camp headquarter; grass fields and shrubby vegetation
	35° 06.25’ W		

Collected sand flies were separated from other insects and placed in labeled vials containing 70% ethanol until processing for morphological identification [12]. For females, the head (containing the cibarium) and the last three abdominal segments (containing the spermathecae) were used for morphological identification, whereas the thorax and the remaining part of the abdomen were used for DNA extraction and PCR testing.

### DNA extraction and *Leishmania* spp. detection

DNA extraction was performed from 1,003 females (grouped in 108 pools) belonging to four species: *Lu*. *choti* (688 females grouped in 71 pools), *Lu*. *sordellii* (197 females grouped in 24 pools), *Lu*. *complexa/wellcomei* (113 females grouped in 12 pools) and *Lu*. *amazonensis* (one pool of five females). Each pool contained 5–10 specimens of the same species and separated according to date and place of collection. DNA extraction was performed using the Chelex 100 resin as described elsewhere [13]. Extracted DNA samples were kept frozen at –20°C until testing.

Real-time PCR testing for detecting *Leishmania* kinetoplast DNA was performed using the primers LEISH-1 (5’-AACTTTTCTGGTCCTCCGGGTAG-3’) and LEISH-2 (5’-ACCCCCAGTTTCCCGCC-3’) and the TaqMan probe FAM-5’-AAAAATGGGTGCAGAAAT-3’-non-fluorescent quencher-MGB, as described elsewhere [14]. All real-time PCR assays were run on a QuantStudio 5 Real-Time PCR machine (Applied Biosystems) and contained a standard curve of 10-fold serial dilutions (1 ng, 100 pg, 10 pg, 1 pg, 100 fg, 10 fg and 1 fg per reaction mixture) from DNA of *L*. *infantum* and a no-template control (DNA-free water). The real-time PCR results were analyzed using QuantStudio Design and Analysis Software v1.4 (Applied Biosystems).

DNA was also extracted from human patients using QIAamp DNA mini kit (Qiagen), according to the manufacturer’s recommendations. Conventional PCR for the detection of *Leishmania* (*Viannia*) DNA in human samples was performed as described elsewhere [15].

### *Leishmania* characterization by restriction enzyme analysis

PCR products (10 μl) were digested with HaeIII (1 μl) (New England BioLabs) for 1 h, at 37°C and then electrophoresed in a 3% high-fidelity agarose gel (Invitrogen). Bands were stained with ethidium bromide. Positive controls (amplified standard DNA from both *L*. *infantum* and *L*. *braziliensis*) were included in each analysis. The species of *Leishmania* detected in each sand fly pool was determined by comparing the banding profile with the ones obtained with positive controls. A 100 bp ladder (Invitrogen) was used as molecular weight.

### Cutaneous leishmaniasis among soldiers

Secondary data on human cases of cutaneous leishmaniasis were obtained from the Reference Service on Leishmaniasis, Aggeu Magalhães Institute, Oswaldo Cruz Foundation, Recife, Brazil. All cases whose patients were soldiers involved in training activities in the study area during 2012–2014 were included in this study. In brief, these patients presented skin lesions suggestive of cutaneous leishmaniasis and were referred to a local military hospital in Recife. At the hospital, the physician in charge determined the diagnosis, initially based on clinic-epidemiological data, and then by leishmanin skin test (Montenegro skin test) and skin-scraping cytology, as recommended by Ministry of Health of Brazil [16]. Skin samples from these patients were seeded in tubes containing biphasic Novy-MacNeal-Nicolle culture medium at 26°C. Isolates obtained were identified using multilocus enzyme electrophoresis [17] at the Oswaldo Cruz Institute (Rio de Janeiro, Brazil) and using monoclonal antibodies [18] at the Evandro Chagas Institute (Belém do Pará, Brazil). Geographical coordinates and altitude of each patient’s house were recorded using a Garmin eTrex Venture HC GPS (Garmin International Ltd, US).

### Meteorological data

Meteorological data (i.e., relative humidity, monthly average temperature, and precipitation) for the whole period of study were obtained from Instituto de Tecnologia de Pernambuco (ITEP) (meteorological station: 82900). The saturation deficit (SD) was calculated as follows: SD = (1 − RH/100) × 4.9463 × *e*^0.0621 × T^, where RH is relative humidity and *T* is temperature.

### Data analysis

The correlation between climatic variables and the number of sand flies collected monthly or daily was done using Spearman’s (*rs*) or Pearson’s (*r*) correlation coefficients, as appropriate. Normality of data was assessed using Lilliefors. The number of sand flies of each species collected according to collection sites or months was compared using Kruskal-Wallis. The significance level was set at *P* < 0.05. Statistical analyses were performed using BioEstat, version 5.3 [19]. The diversity indices (Species richness, Shannon’s diversity index and Pielou’s equitability index) and abundance were calculated using PAST, version 2.16 [20]. The standardized index of species abundance (SISA) was as described elsewhere [21].

## Results

### Sand fly species diversity

A total of 24,606 sand flies (Table 2), being 12,683 males and 11,923 females (overall sex ratio close to unity), belonging to 25 species (i.e., *Lutzomyia choti*, *Lutzomyia longispina*, *Lutzomyia complexa*, *Lutzomyia sordellii*, *Lutzomyia amazonensis*, *Lutzomyia walkeri*, *Lutzomyia wellcomei*, *Lutzomyia quinquefer*, *Lutzomyia evandroi*, *Lutzomyia barrettoi barrettoi*, *Lutzomyia ayrozai*, *Lutzomyia capixaba*, *Lutzomyia naftalekatzi*, *Lutzomyia claustrei*, *Lutzomyia schreiberi*, *Lutzomyia umbratilis*, *Lutzomyia whitmani*, *Lutzomyia brasiliensis*, *Lutzomyia viannamartinsi*, *Lutzomyia shannoni* complex, *Lutzomyia yuilli pajoti*, *Lutzomyia aragaoi*, *Lutzomyia furcata*, *Lutzomyia migonei* and *Lutzomyia oswaldoi*) were identified.

**Table 2:** Sand fly species collected according to sex and Standardized Index of Species Abundance (SISA), July 2012–July 2014.

Species	Males		Females		Total		SISA
	*n*	%	*n*	%	*n*	%	
*Lu*. *amazonensis*	102	0.80%	141	1.29%	243	1.03%	0.78
*Lu*. *aragaoi*	0	0.00%	1	0.01%	1	0.00%	0.03
*Lu*. *ayrozai*	2	0.02%	46	0.42%	48	0.20%	0.36
*Lu*. *barrettoi barrettoi*	3	0.02%	46	0.42%	49	0.21%	0.29
*Lu*. *brasiliensis*	1	0.01%	5	0.05%	6	0.03%	0.14
*Lu*. *choti*	9,990	78.77%	7,961	72.58%	17,951	75.90%	1.00
*Lu*. *capixaba*	4	0.03%	36	0.33%	40	0.17%	0.49
*Lu*. *claustrei*	14	0.11%	17	0.15%	31	0.13%	0.43
*Lu*. *complexa*	356	2.81%	0	0.00%	356	1.51%	0.73
*Lu*. *evandroi*	28	0.22%	68	0.62%	96	0.41%	0.53
*Lu*. *furcata*	1	0.01%	0	0.00%	1	0.00%	0.03
*Lu*. *longispina*	1,622	12.79%	1,794	16.36%	3,416	14.44%	0.93
*Lu*. *migonei*	1	0.01%	0	0.00%	1	0.00%	0.01
*Lu*. *naftalekatzi*	20	0.16%	14	0.13%	34	0.14%	0.42
*Lu*. *sordellii*	266	2.10%	613	5.59%	879	3.72%	0.89
*Lu*. *oswaldoi*	1	0.01%	0	0.00%	1	0.00%	0.04
*Lu*. *quinquefer*	12	0.09%	108	0.98%	120	0.51%	0.58
*Lu*. *schreiberi*	7	0.06%	12	0.11%	19	0.08%	0.25
*Lu*. *shannoni* complex	1	0.01%	3	0.03%	4	0.02%	0.13
*Lu*. *walkeri*	109	0.86%	77	0.70%	186	0.79%	0.73
*Lu*. *wellcomei*	130	1.02%	0	0.00%	130	0.55%	0.55
*Lu*. *whitmani*	11	0.09%	4	0.04%	15	0.06%	0.19
*Lu*. *umbratilis*	0	0.00%	16	0.15%	16	0.07%	0.10
*Lu*. *viannamartinsi*	2	0.02%	3	0.03%	5	0.02%	0.10
*Lu*. *yuilli pajoti*	0	0.00%	3	0.03%	3	0.01%	0.04
Total	12,683	53.63%	10,968	46.37%	23,651^a^	100.00%	NA

^a^ Females identified as *Lutzomyia complexa*/*wellcomei* (*n* = 955) are not included in this table.

NA: not applicable; *Lu*.: *Lutzomyia*.

*Lutzomyia choti*, *Lu*. *longispina*, *Lu*. *complexa* and *Lu*. *sordellii* were the most frequently collected species, representing together 95.8% of the total collections. Yet, *Lu*. *barrettoi barrettoi*, *Lu*. *ayrozai*, *Lu*. *capixaba*, *Lu*. *naftalekatzi*, *Lu*. *claustrei*, *Lu*. *schreiberi*, *Lu*. *umbratilis*, *Lu*. *whitmani*, *Lu*. *brasiliensis*, *Lu*. *viannamartinsi*, *Lu*. *shannoni* complex, *Lu*. *yuilli pajoti*, *Lu*. *aragaoi*, *Lu*. *furcata*, *Lu*. *migonei and Lu*. *oswaldoi* were rarely collected, representing together 1.1% of the total.

The species richness was higher in CS2 (20 spp.), followed by CS1 (17 spp.), CS3 (17 spp.), CS8 (16 spp.) and CS9 (16 spp.), respectively. The highest Shannon’s diversity and Pielou’s equitability indexes were found in CS9 and CS6 (Table 3). Overall, no significant difference was found in the number of specimens from each species in relation to the site of collection (H = 11.874; df = 9, *P* = 0.221).

**Table 3:** Species richness (S), Shannon’s diversity index (H’) and Pielou’s equitability index (J) according to collection site in the Atlantic Forest fragment, northeastern Brazil.

Collection site	Richness (S)	Shannon’s diversity index (H')	Pielou’s equitability index (J)
CS1	17	0.8588	0.3031
CS2	20	0.6733	0.2248
CS3	17	0.8297	0.2928
CS4	12	0.9518	0.383
CS5	15	0.82	0.3028
CS6	13	1.079	0.4209
CS7	15	1.076	0.3974
CS8	16	1.083	0.3904
CS9	16	1.254	0.4524
CS10	11	0.6667	0.2781

### Sand fly temporal dynamics and climate variables

**Fig 2:**
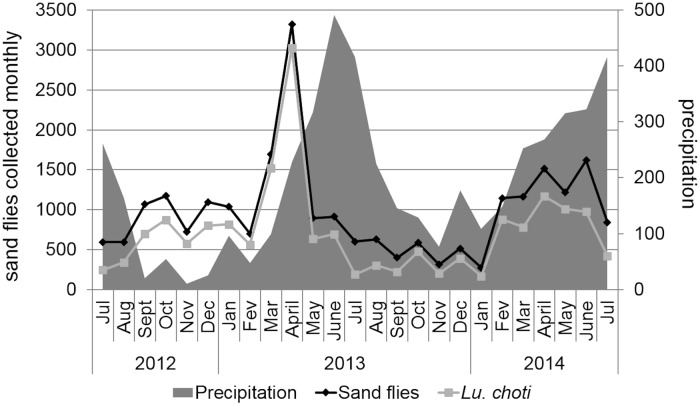
Monthly number of sand flies collected, July 2012–July 2014. Average monthly precipitation is also depicted.

Overall, the sand fly population studied herein presented a unimodal temporal distribution pattern, peaking in the first semester of each year. The number of sand flies collected monthly during the whole study period ranged from 271 to 3,324, peaking in March (1,691) and April (3,324) 2013 and April (1,512), May (1,217) and June (1,619) 2014 (Fig 2). No significant variation was found in the number of specimens from each species in relation to the month of collection (H = 14.404; df = 24, *P* = 0.937).

**Fig 3:**
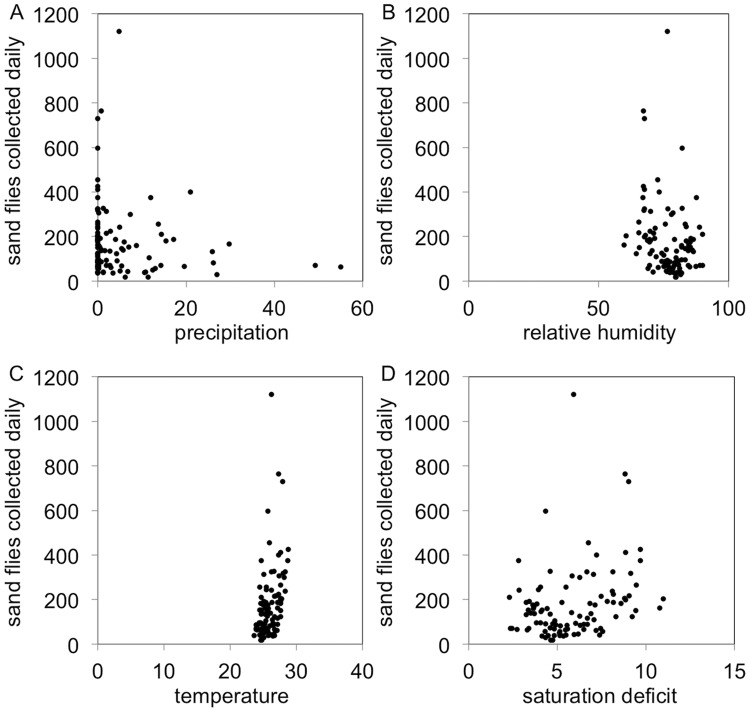
Scatter plot with the number of *Lutzomyia choti* captured versus climatic variables. (A) Precipitation. (B) Relative humidity. (C) Temperature. (D) Saturation deficit.

No correlation was found between the monthly number of sand flies collected and climate variables (*rs* = 0.29, *P* = 0.167, for temperature; *rs* = 0.14, *P* = 0.519, for precipitation; *rs* = –0.05, *P* = 0.829, for relative humidity; and *rs* = 0.11, *P* = 0.608, for saturation deficit). However, comparing daily data, the number of sand flies collected was significantly correlated with temperature (*rs* = 0.29, *P* = 0.003), but not with precipitation (*rs* = –0.185, *P* = 0.066), relative humidity (*rs* = –0.153, *P* = 0.128) and saturation deficit (*rs* = 0.181, *P* = 0.072). Considering only the most abundant species (i.e., *Lu*. *choti*), the daily number of specimens collected was significantly correlated with temperature (*rs* = 0.44, *P* < 0.001), precipitation (*rs* = –0.242, *P* = 0.015), relative humidity (*rs* = –0.25, *P* = 0.012) and saturation deficit (*rs* = 0.293, *P* = 0.003) (Fig 3).

### Cutaneous leishmaniasis among soldiers

Forty male soldiers (age range, 19–22 years) were diagnosed with cutaneous leishmaniasis in a regional military hospital from January 2012 to December 2014. Most of the soldiers were involved in nocturnal training activities in the study area in September 2013 (*n* = 7), October 2013 (*n* = 16), and September 2014 (*n* = 11). Among the remaining soldiers, two were training in June 2013, two in July 2013, one in September 2011 and one in October 2011. One of the soldiers that were training in September 2013 was also in the forest in June 2013.

Most of them were diagnosed with cutaneous leishmaniasis in November (*n* = 22), December (*n* = 10) or October (*n* = 6); the remaining two cases were diagnosed with cutaneous leishmaniasis in January 2012. All suspected cases but three were confirmed by one or more diagnostic test: 100% (30/30; eight refused to do the test) were positive at the leishmanin skin test, 31.6% (12/38) at cytology, 28.9% (11/38) at PCR, and 21.1% (8/38) at culture. All eight isolates obtained from those patients were all characterized as *L*. *braziliensis*.

Patients presented localized ulcers on legs, forearm, hands and neck. In most of the cases (84.2%), the diagnosis was made 1–2 months after the training period in the forest. All patients were successfully treated with n-methyl-glucamine antimoniate (Glucantime), except one whose lesion healed spontaneously without treatment.

### *Leishmania* detection by real-time PCR and characterization by restriction enzyme analysis

**Fig 4:**
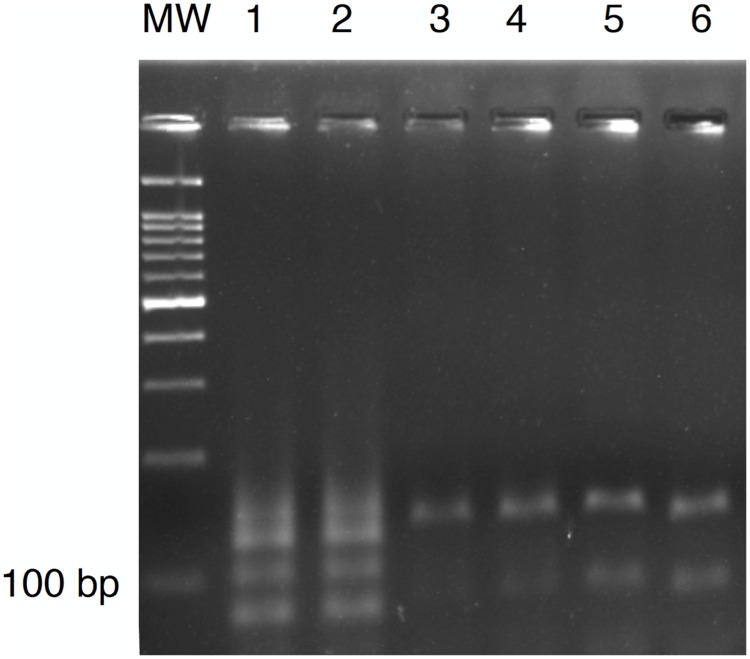
Restriction enzyme (HaeIII) analysis. MW, Molecular weight (100 bp); 1–2, *Leishmania infantum* (positive control); 3–4, *Leishmania braziliensis* (positive control); 5–6, F968.

Out of 1,003 females tested, six pools (10 females each) of *Lu*. *choti* were positive at real-time PCR, which gives an overall minimum infection rate of 0.6%. Considering only *Lu*. *choti*, the minimum infection rate was 0.87% (6/688). Three pools (i.e., F968, F651, and F1002) presented a HaeIII restriction profile identical to *L*. *braziliensis* (Fig 4). The three remaining positive pools did not show any pattern probably due to the very low amount of DNA detected by real-time PCR.

## Discussion

A high diversity of sand flies was recorded in the studied Atlantic forest remnant studied herein. Our results, together with data available in the literature [11, 22, 23], suggest that most sand fly species found in Atlantic forest remnants are still more adapted to the forest than to human-modified environments; i.e., only 11 out 25 species identified in the study were collected in the animal stable, and typically in low numbers. Indeed, in some areas where cutaneous leishmaniasis by *L*. *braziliensis* is endemic, some vector species appear to be almost exclusively found in the forest interior or forest edge, rather than in the peridomicile [11, 22, 23]. Nonetheless, it is acknowledged that some vector species like *Lu*. *whitmani* are well adapted to human-dwellings [11]. This is in agreement with data obtained in the current study, where *Lu*. *whitmani* was found in very low numbers (15 specimens in the whole study period) and mostly in the horse stable (66.7% of the specimens collected).

The sand fly population studied herein presented a well-defined unimodal temporal distribution pattern, peaking in the first semester of each year. The highest peak was recorded in April 2013, with over 3,324 sand flies collected. This finding is congruent with a preliminary 1-year study conducted in 2003 in the same area [8]. These results clearly indicate that this sand fly population displays a relatively stable temporal distribution pattern, throughout the years. In turn, this suggests that the studied Atlantic forest remnant has not been much modified during the last 10 years or that possible modification that may have occurred during this period did not influence the sand fly population, neither negatively nor positively.

Still regarding the population dynamics, it is important to state that the heavy rains observed during some capture nights may have reduced the efficiency of our light traps during these nights. While obvious, this is not usually considered in the analysis of studies on sand fly population dynamics. It is crucial to consider this aspect because an apparent decline in the population may not be a real decline but merely an artifactual reduction of collection due to heavy rains or strong winds occurring during trapping nights. For instance, the reduced number of sand flies collected during some months (e.g., June and July 2013) may be a result of heavy rains recorded in these months. So, the common assertion that sand fly populations present a peak after rainy periods may be an artifact in some Brazilian regions. Indeed, it is expected that in drier areas sand flies (e.g., *Lu*. *longipalpis*) may be more active after rainy periods, when the relative humidity becomes higher [24]. However, in Atlantic forest remnants, where environmental conditions are optimal to sand flies throughout the year, population peaks after rainy periods may be actually due to adults that resume their activity after periods of unfavorable weather conditions rather than due to the emergence of new adults. What do adult sand flies do when it rains is uncertain. Small fliers appear to be more robust than we think to in-flight perturbations [25]. Mosquitoes, which are larger than sand flies, have a strong exoskeleton and low mass that make them impervious to falling drops [25]. Water resistant hairs cover the wings and the whole body of sand flies and this may also protect them from falling raindrops.

*Lutzomyia choti* was the most abundant species and its daily number was positively correlated with temperature and saturation deficit, which is in line with a recent investigation conducted in low-density residential rural area, with mixed forest/agricultural exploitation [11]. This sand fly species was the only one found infected by *Leishmania* in the present study, with an estimated minimum infection rate of 0.87%. Real-time PCR-positive females were collected in January (one pool), February (two pools), April (one pool) and June (two pools). Whether by coincidence or not, all infected pools where collected in the first semester of the year, in parallel to the main sand fly population peak recorded in this study. *Lutzomyia choti* is one of the most common sand fly species in Atlantic forest remnants in Brazil [8, 10, 11]. It is also willing to feed on humans [9]. Altogether, these findings may indicate that *Lu*. *choti* may be an important vector of *L*. *braziliensis* in remnants of Atlantic forest in Brazil.

The period of the year when there is highest risk of *Leishmania* spp. transmission is always an issue of debate. In fact, one may say that the risk is higher when the sand fly population peaks up; higher biting rates. Other may argue that the highest risk would be later, when the vector population drops down and gets older; lower biting rates but higher infection rates in sand flies. Indeed, after emergence from the pupae, adult females need to take a meal to become infected, mature the infection in its gut to be able to transmit the parasites to a susceptible host. Taking into account our data on sand fly temporal distribution pattern and *Leishmania* infections in both sand flies and soldiers, we may speculate that the risk of cutaneous leishmaniasis may be permanent, but probably higher in the second semester. Nonetheless, this is a hypothesis that needs further investigations to be confirmed.

Cases of cutaneous leishmaniasis have been sporadically detected in the study area [3–5]. These cases are usually associated to soldiers or other military personnel that were involved in nocturnal activities in the forested environment. Indeed, the risk of infection by *L*. *braziliensis* in Atlantic forest remnants is eminent, as sand fly vectors [7–11] and reservoirs (e.g., small rodents) of this parasite are abundant in this biome [26–28]. It means that anyone overnighting in Atlantic forest remnants should seriously consider the adoption of protective measures, such as the use of repellents on bare skin or clothes and insecticide-treated tents. This should help reducing the risk of cutaneous leishmaniasis, especially among individuals like soldiers and forest workers that cannot avoid the contact with forested environments during the night.

The Atlantic forest biome has been reduced to almost nothing of its original land cover [29]. Nonetheless, this biome is still home to a great diversity of animals and plants. Among animals living in Atlantic forest remnants, there are arthropods that may act as vectors and small mammals that may act as reservoirs of disease agents (e.g., *Leishmania* spp. and *Rickettsia* spp.). It has been demonstrated that habitat fragmentation and biodiversity loss can increase the risk of pathogen transmission to humans through the so-called dilution effect [30–32]. It is yet to be investigated whether the almost complete destruction of the Atlantic forest biome has played a role on the epidemiology of cutaneous leishmaniasis in Brazil.

## Concluding remarks

This study demonstrates that the temporal dynamics of sand flies is correlated to some extent to climate variables, with some species contrasts. The finding of *Lu*. *choti* females infected with *L*. *braziliensis*, along with its known anthropophyly and high abundance in Atlantic forest fragments in Brazil, highlight the need for further studies to assess the vector competence of this sand fly for transmitting *L*. *braziliensis* under experimental conditions. Finally, people overnighting in Atlantic rainforest remnants should adopt preventative measures such as the use of repellents on bare skin or clothes and insecticide-treated tents to reduce their exposure to sand flies and other potential disease vectors.
